# Supplementary material for: An Integrative Analysis of the Immune Features of Inactivated SARS-CoV-2 Vaccine (CoronaVac)
Source: Vaccines (Basel). 2022 May 30;10(6):878. doi: 10.3390/vaccines10060878 (PMC9231306; doi:10.3390/vaccines10060878)
Supplement: Supplementary file 1 [file vaccines-10-00878-s001.zip › vaccines-1723111-supplementary.pdf]

Figure S1

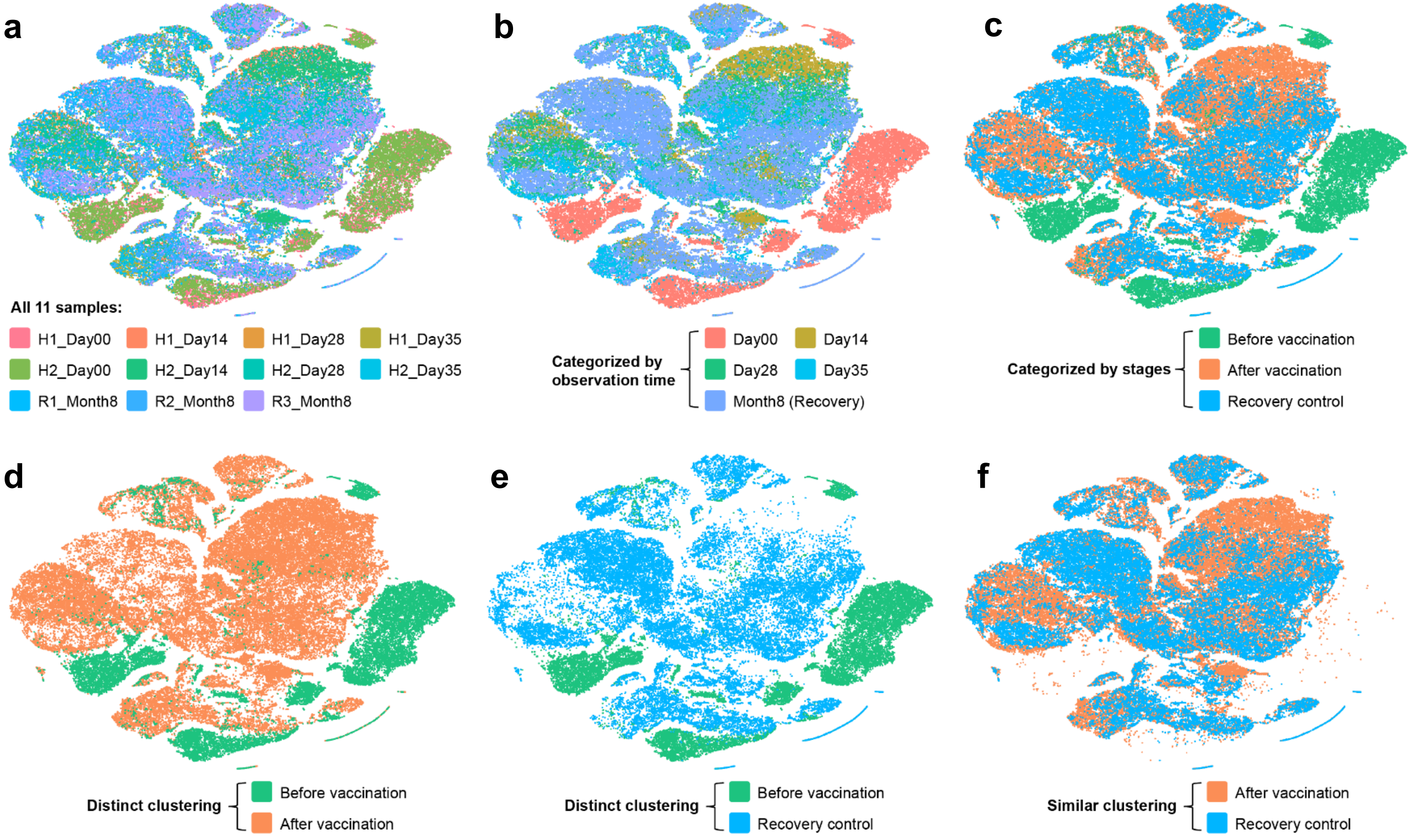

Figure S1. scRNA-seq of PBMC. (a-c) The t-SNE plots of all 103,931 analyzed cells that were categorized and colored according to sample name, observation time and three stages. Day14, Day28, and Day35 can be aggregated into one stage (i.e., after vaccination). (d-f) The t-SNE plots of paired comparisons between three main stages.

Figure S2

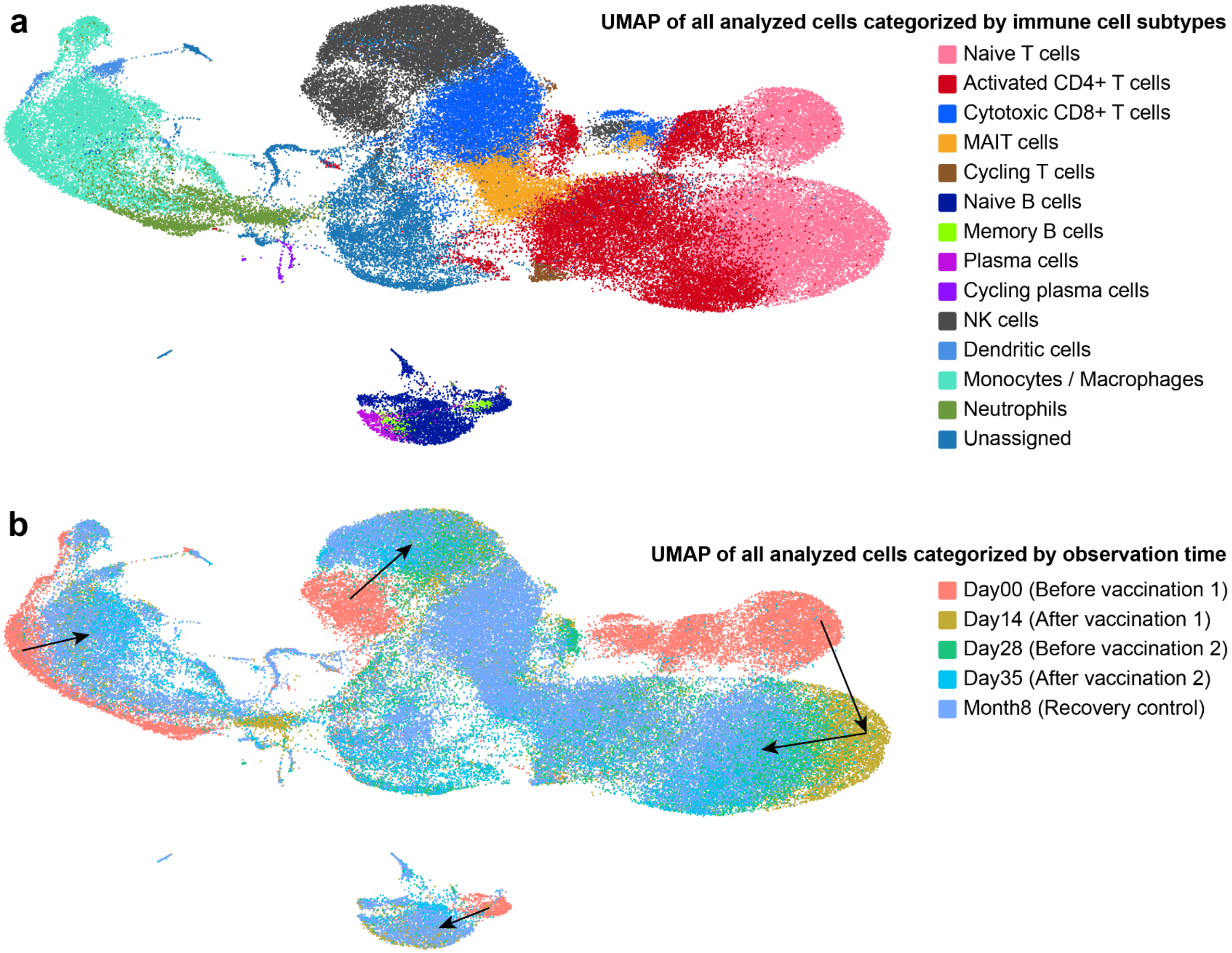

Figure S2. UMAP of all cells analyzed by scRNA-seq. With the UMAP dimension reduction, the 103,931 points or cells from 11 samples were categorized and colored by the immune cell subtypes (**a**) or observation time (**b**). The PBMC transcriptomics of unvaccinated healthy participants (Day00) resulted in distinct distributions from those of vaccinated timepoints (Day14, Day28, and Day35) and the COVID-19 recovery control (Month8) on the UMAP, which was consistent to the t-SNE graph. Immune cell subtypes were identified by a set of gene markers as follows: naïve T cells (CD3G$^+$CCR7$^+$GZMA$^-$), activated CD4$^+$ T cells (CD3G$^+$CD4$^+$IL7R$^+$), cytotoxic CD8$^+$ T cells (CD3G$^+$CD8B$^+$GZMA$^+$), MAIT cells (CD3G$^+$SLC4A10$^+$), cycling T cells (CD3G$^+$MKI67$^+$), naïve B cells (MS4A1$^+$IGHG1$^-$), memory B cells (MS4A1$^+$IGHG1$^+$), plasma cells (MZB1$^+$IGHG1$^+$), cycling plasma cells (MZB1$^+$IGHG1$^+$MKI67$^+$), NK cells (KLRF1$^+$CD3G$^-$), dendritic cells (CD1C$^+$LYZ$^+$; CLEC9A$^+$; LAMP3$^+$; LILRA4$^+$), monocytes (LYZ$^+$CD68$^+$; LYZ$^+$CD14$^+$), macrophages (LYZ$^+$CCL3L1$^+$), and neutrophils (LYZ$^+$IL1B$^+$CXCR4$^-$CST7$^+$).

Figure S3

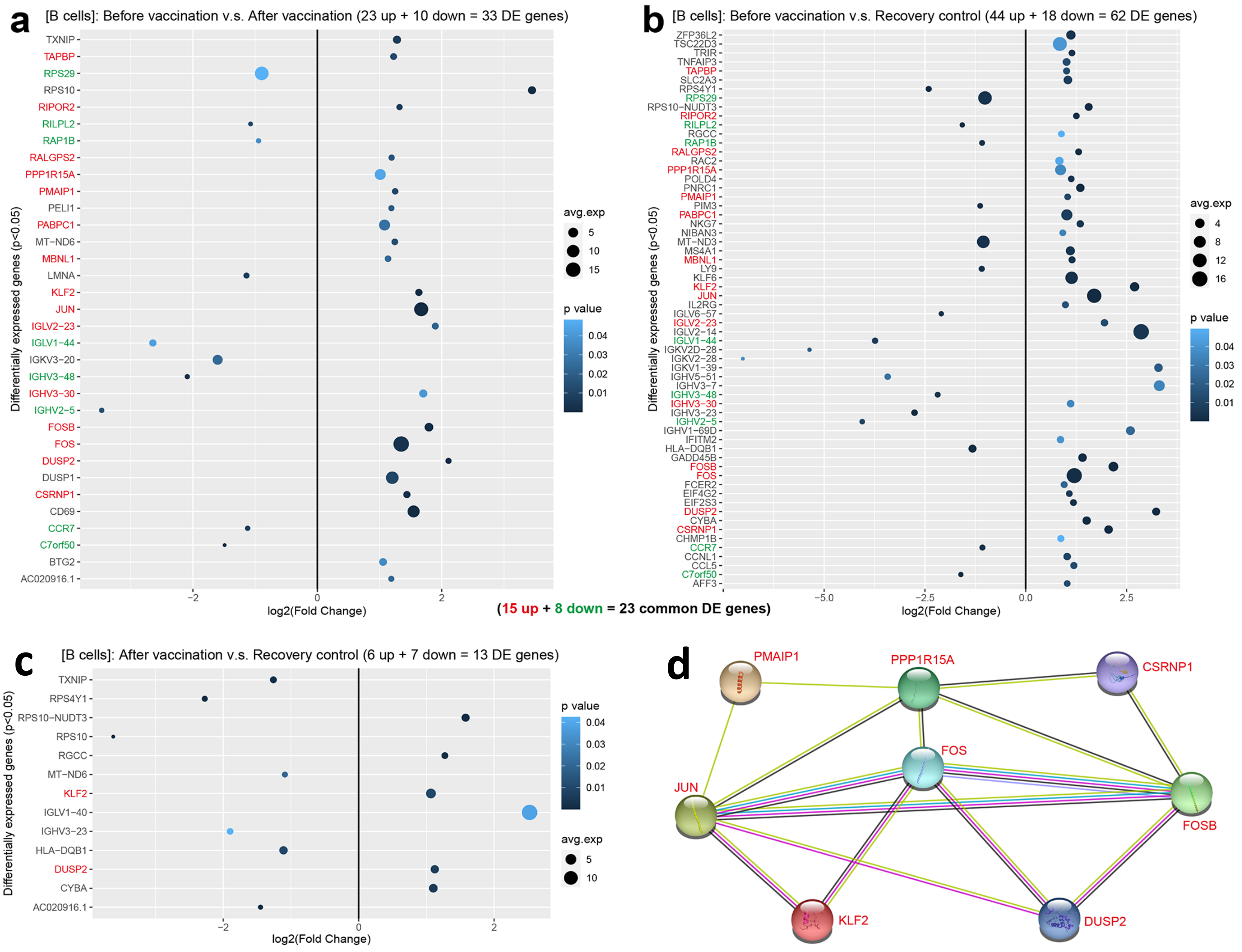

Figure S3. DE genes in B cells. The log2(fold change), p value and average expression of DE genes in B cells were plotted for before vaccination versus after vaccination (a), before vaccination versus recovery control (b), and after vaccination versus recovery control (c). Red fonts for common up-regulated genes in the latter group of each comparison, while green fonts for common down-regulated ones. Among the 23 common DE genes, eight up-regulated genes formed an interaction network (d) according to the STRING database.

Figure S4

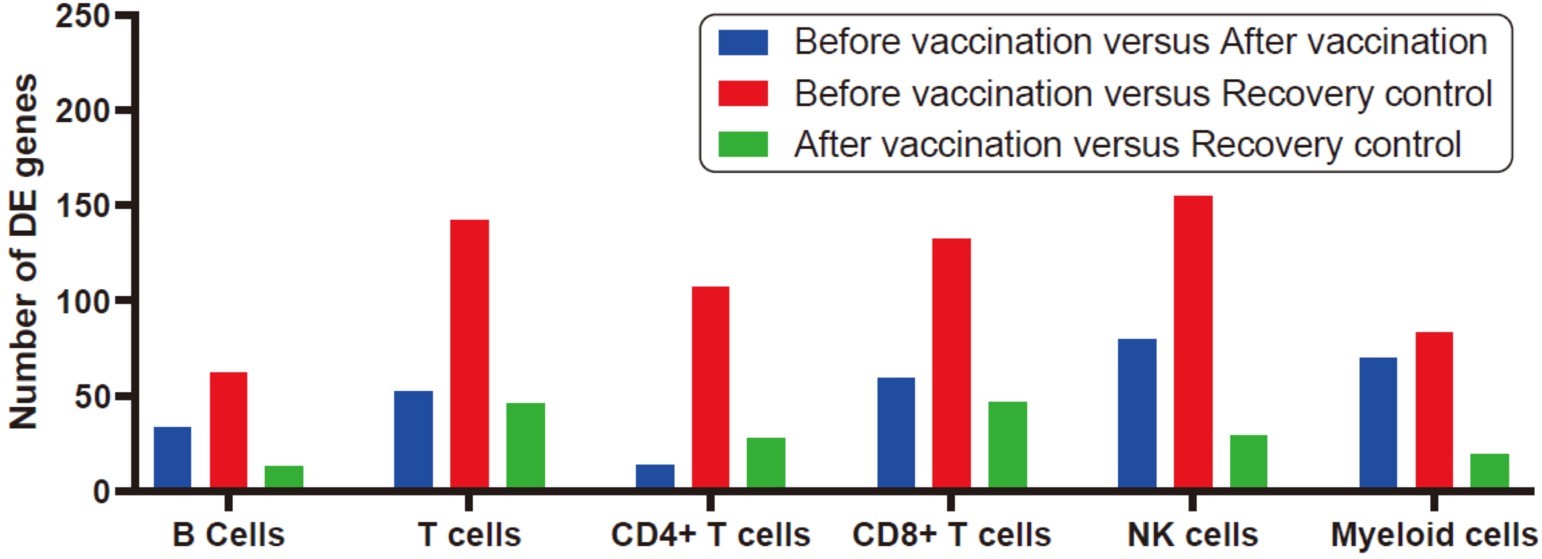

Figure S4. Summary of the DE gene numbers in different immune cells. For most cell types except CD4+ T cells, the fewest DE genes were observed in after vaccination versus recovery control among the three comparisons, indicating that the state after vaccination resembled the COVID-19 recovery control more than that before vaccination.

Figure S5

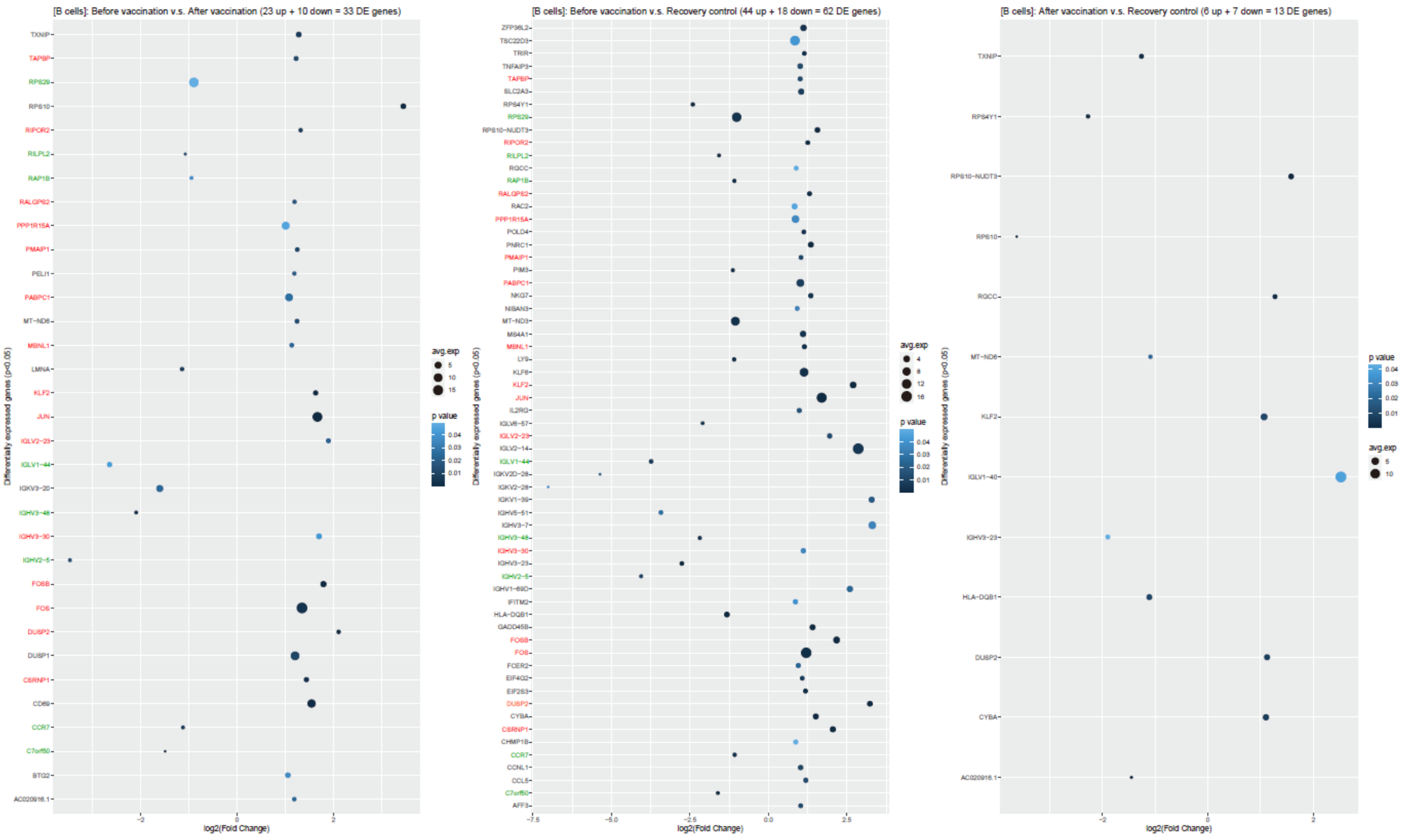

Figure S5. Differentially expressed genes in B cells. Left panel for the comparison of before vaccination versus after vaccination. Middle panel for the comparison of before vaccination versus recovery control. Right panel for the comparison of after vaccination versus recovery control.

Figure S6

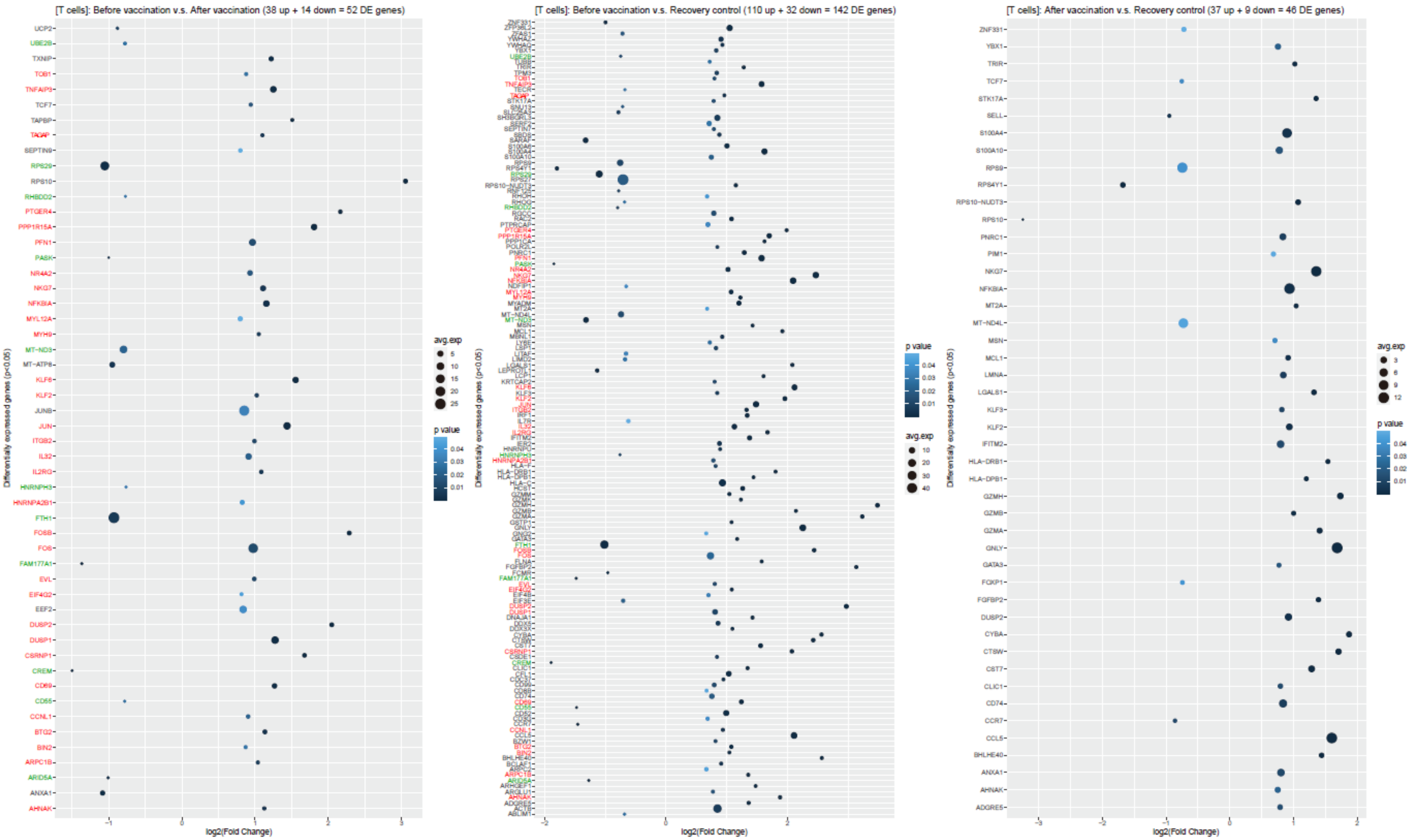

Figure S6. Differentially expressed genes in T cells. Left panel for the comparison of before vaccination versus after vaccination. Middle panel for the comparison of before vaccination versus recovery control. Right panel for the comparison of after vaccination versus recovery control.

Figure S7

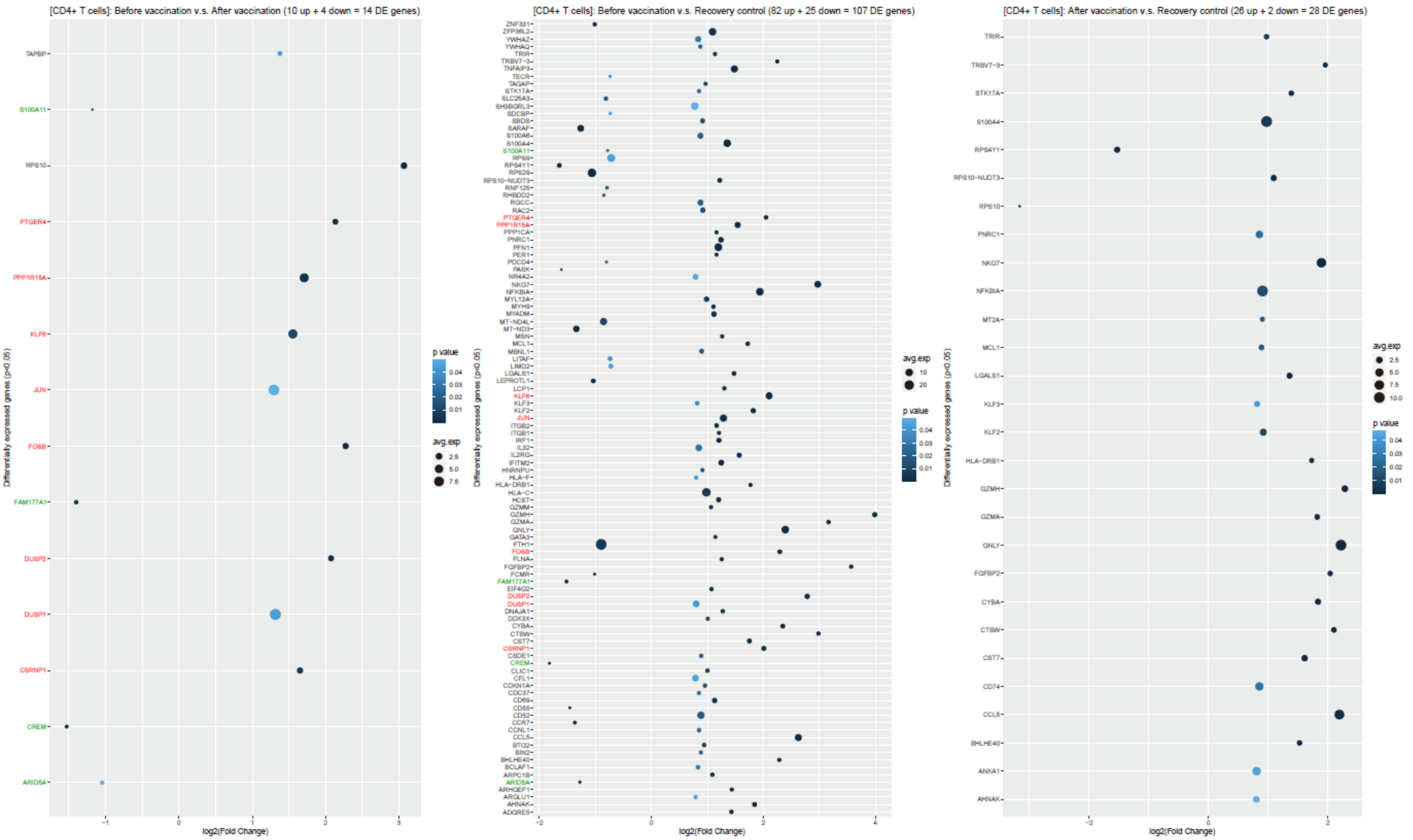

Figure S7. Differentially expressed genes in CD4+ T cells. Left panel for the comparison of before vaccination versus after vaccination. Middle panel for the comparison of before vaccination versus recovery control. Right panel for the comparison of after vaccination versus recovery control.

Figure S8

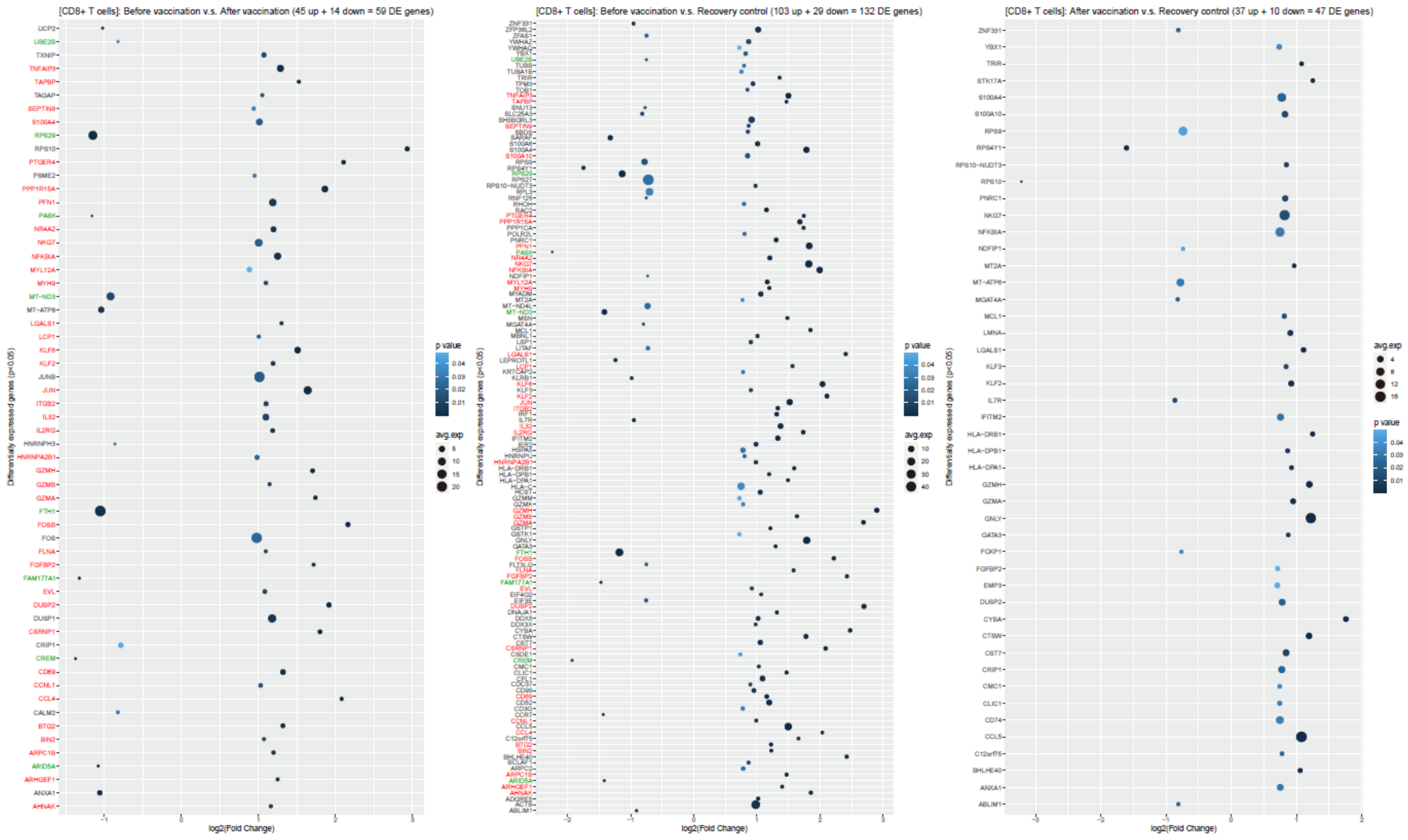

Figure S8. Differentially expressed genes in CD8+ T cells. Left panel for the comparison of before vaccination versus after vaccination. Middle panel for the comparison of before vaccination versus recovery control. Right panel for the comparison of after vaccination versus recovery control.

Figure S9

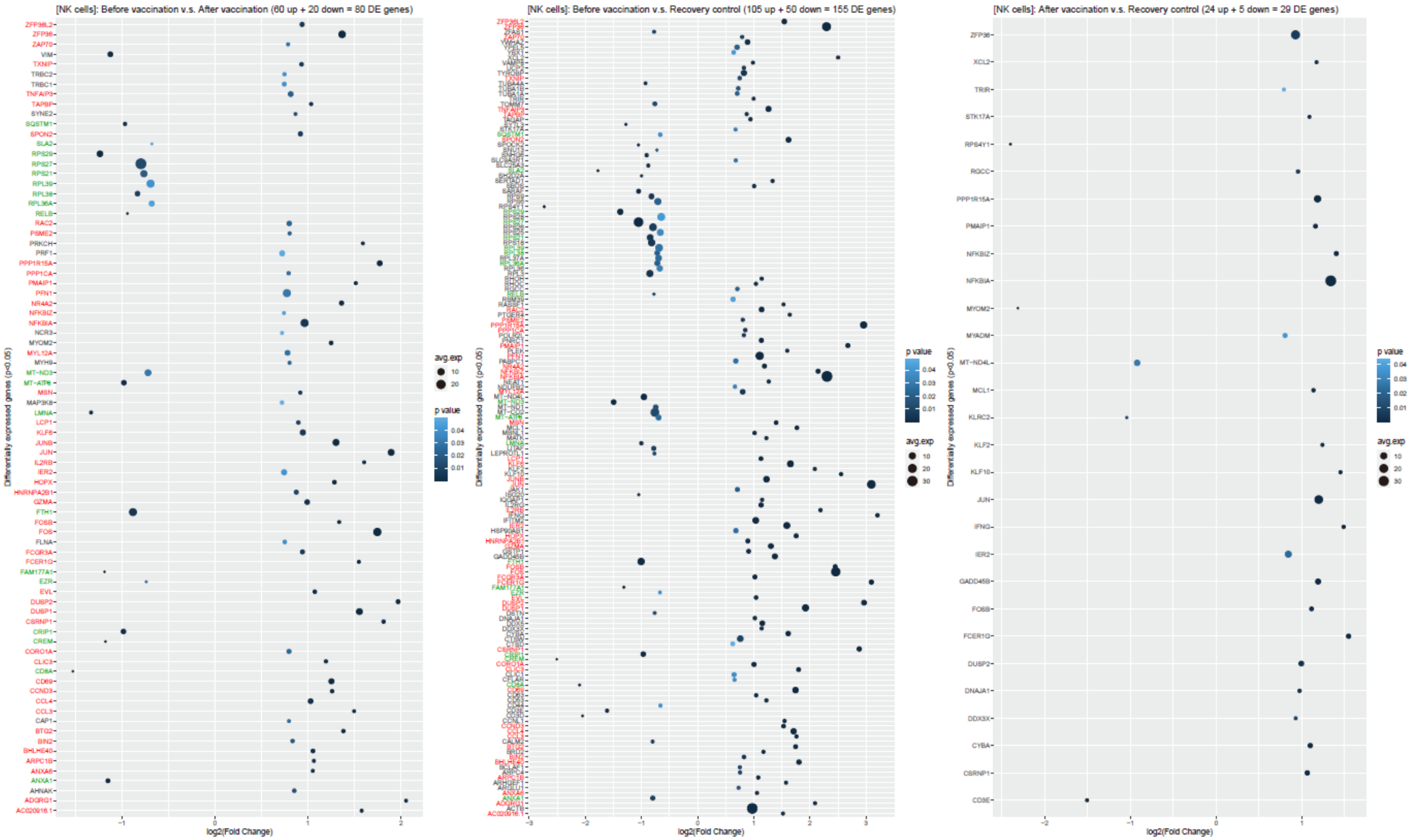

Figure S9. Differentially expressed genes in NK cells. Left panel for the comparison of before vaccination versus after vaccination. Middle panel for the comparison of before vaccination versus recovery control. Right panel for the comparison of after vaccination versus recovery control.

Figure S10

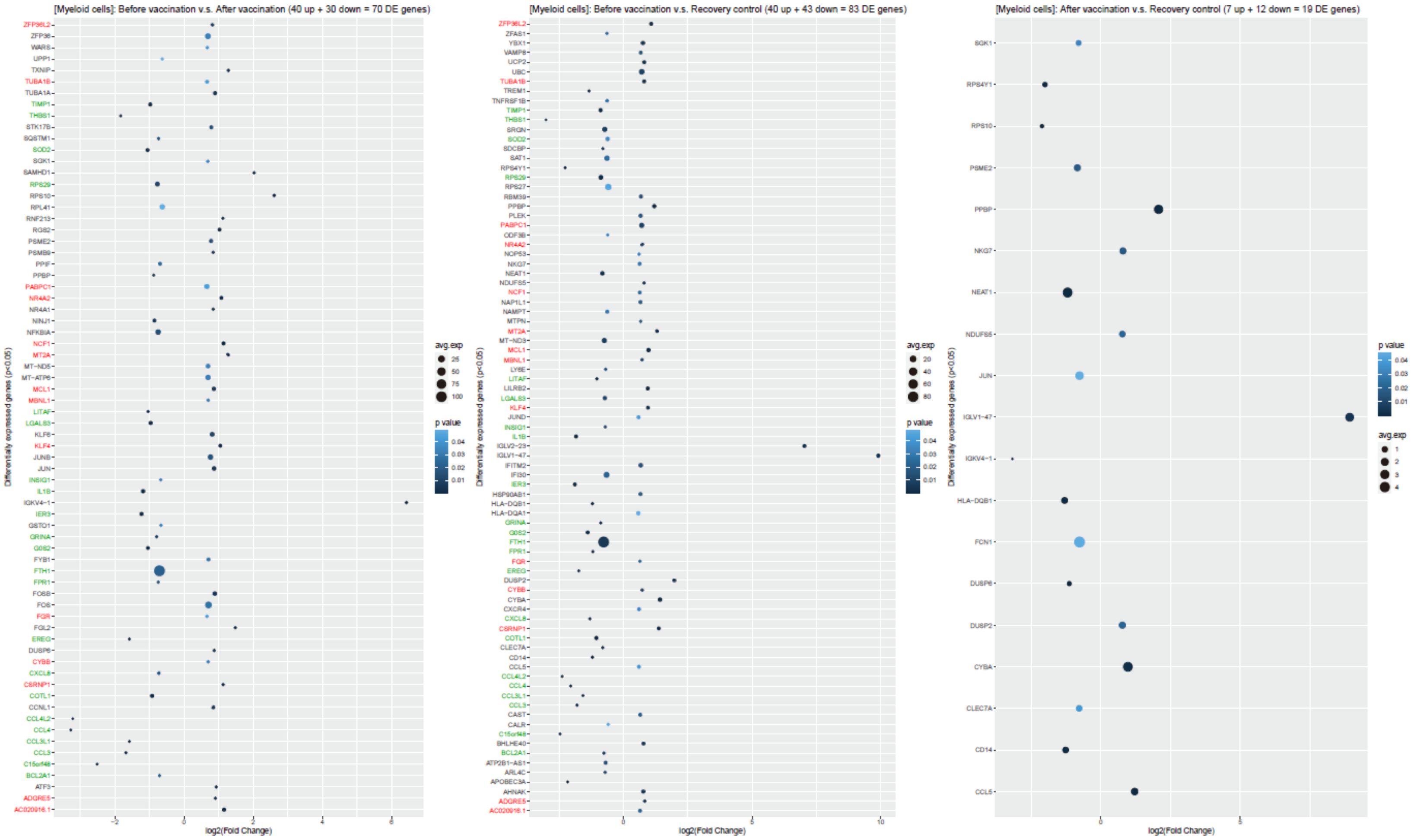

Figure S10. Differentially expressed genes in myeloid cells. Left panel for the comparison of before vaccination versus after vaccination. Middle panel for the comparison of before vaccination versus recovery control. Right panel for the comparison of after vaccination versus recovery control.

Figure S11

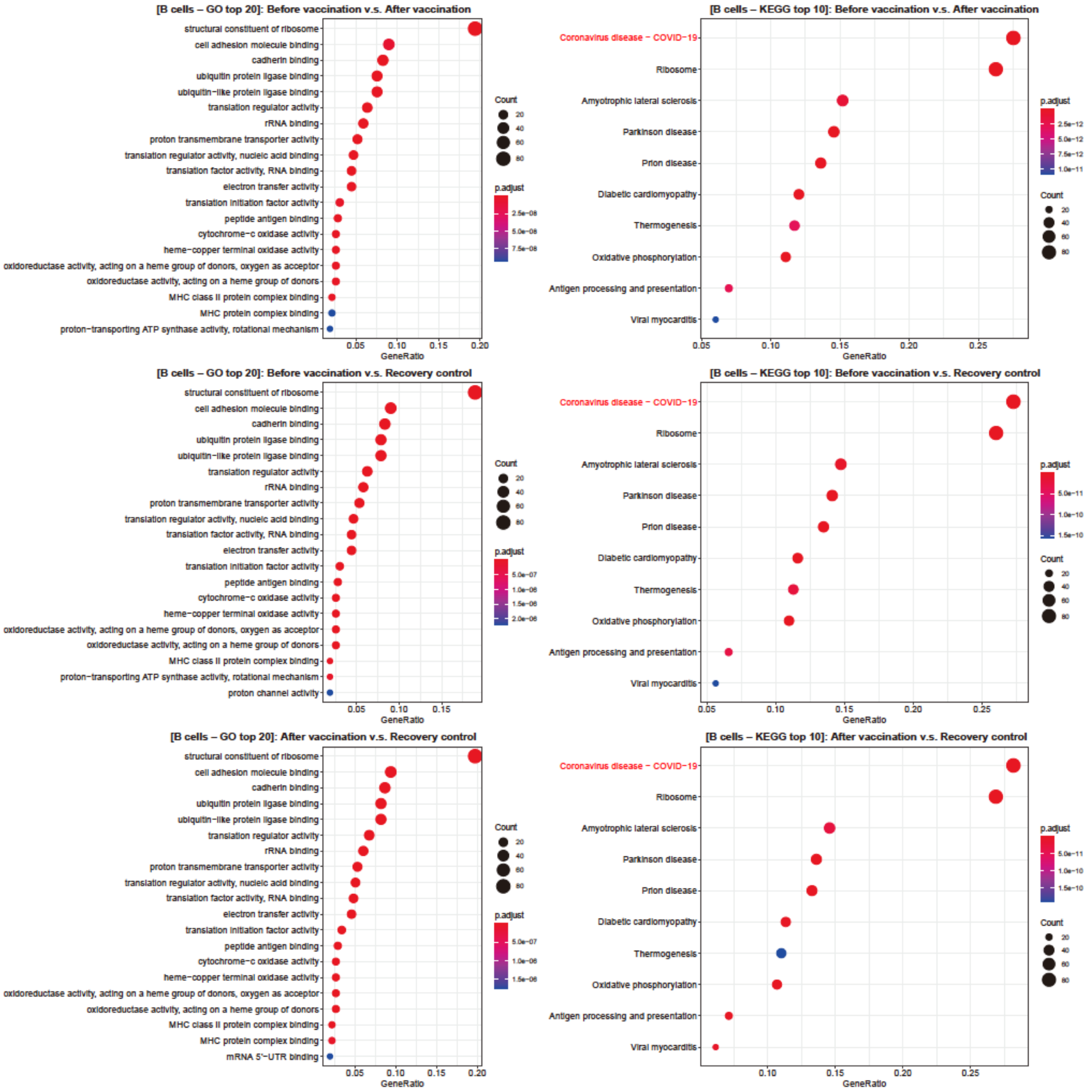

Figure S11. Enrichment GO (left panels) and KEGG (right panels) analyses for B cells.

Figure S12

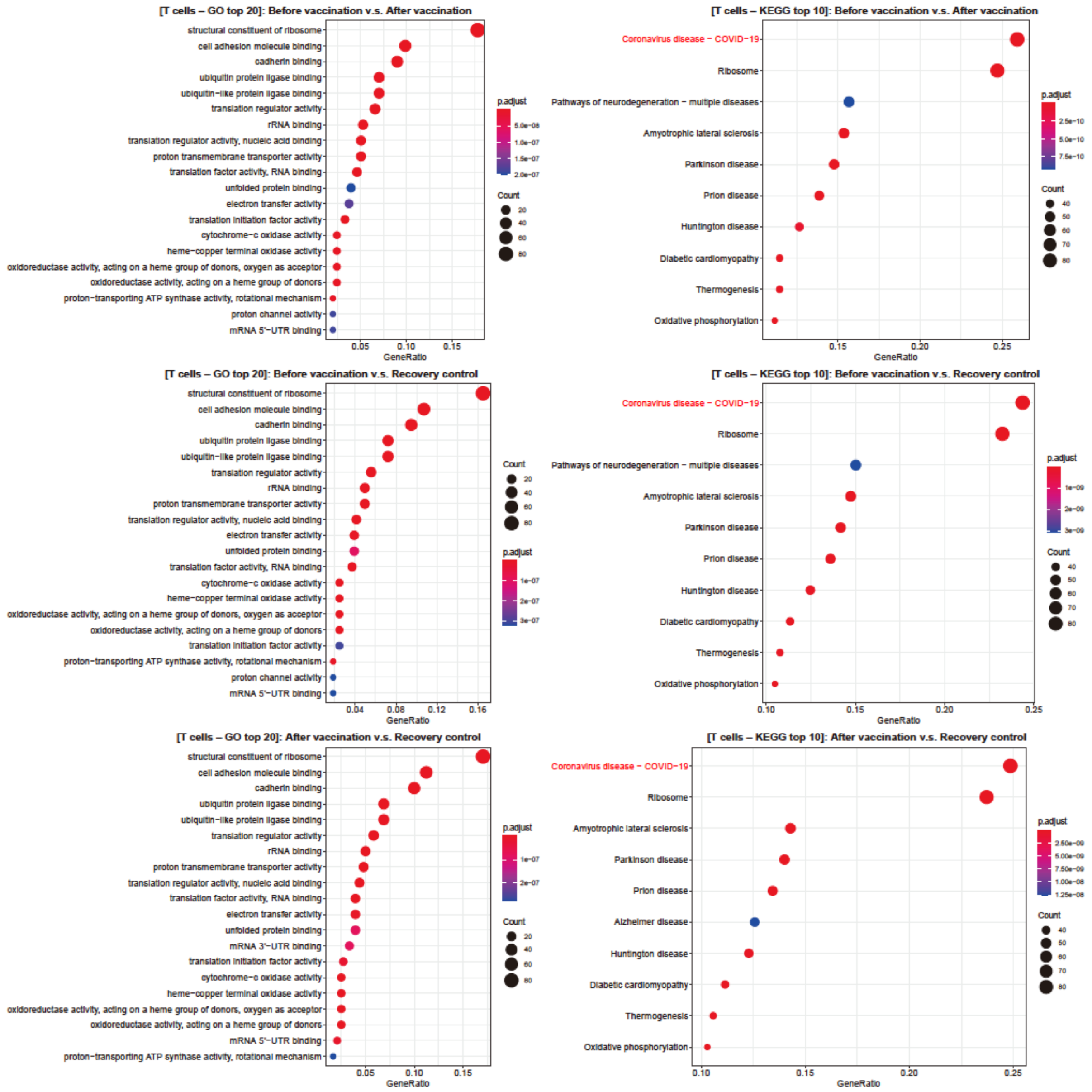

Figure S12. Enrichment GO (left panels) and KEGG (right panels) analyses for T cells.

Figure S13

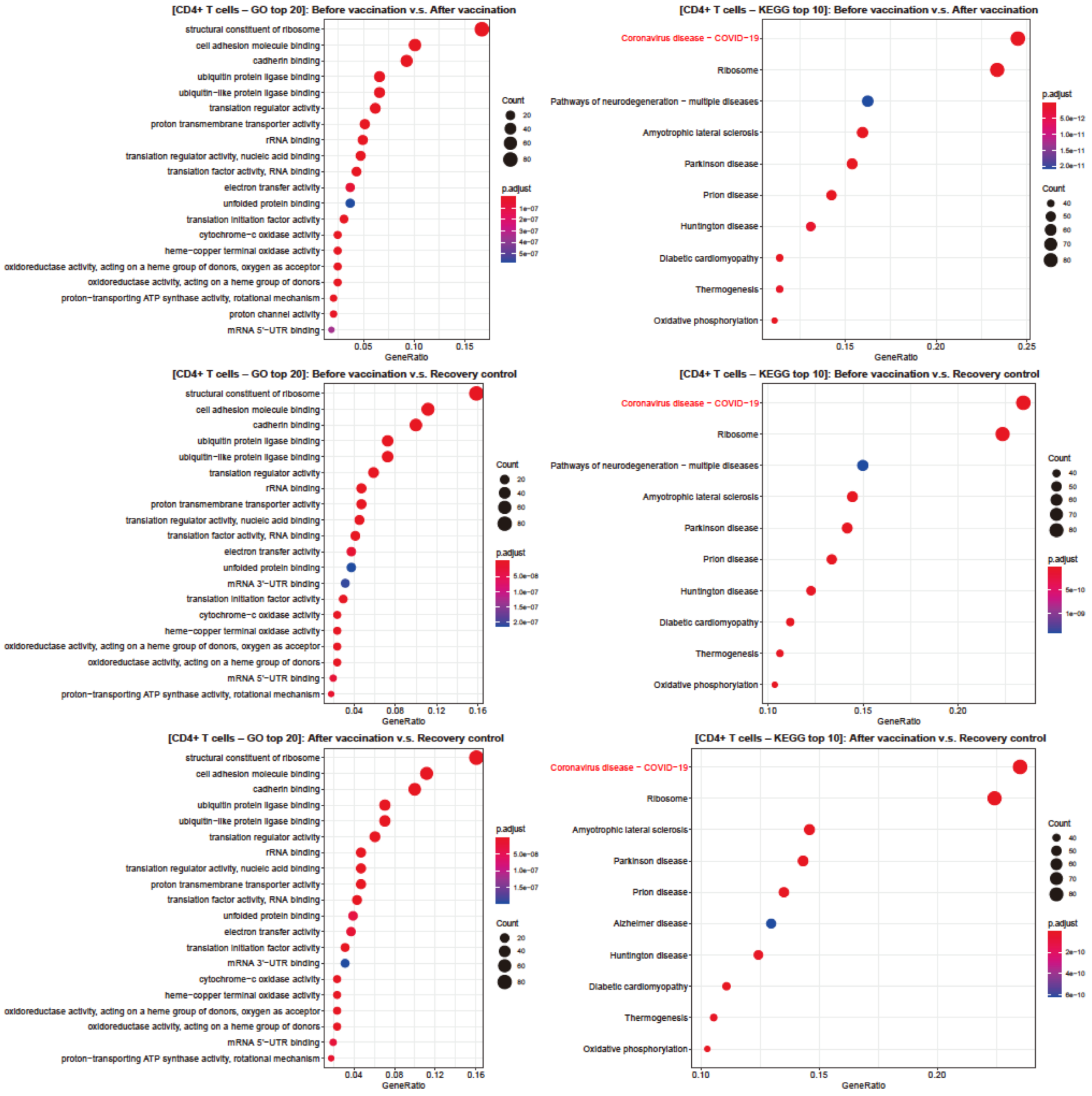

Figure S13. Enrichment GO (left panels) and KEGG (right panels) analyses for CD4+ T cells.

Figure S14

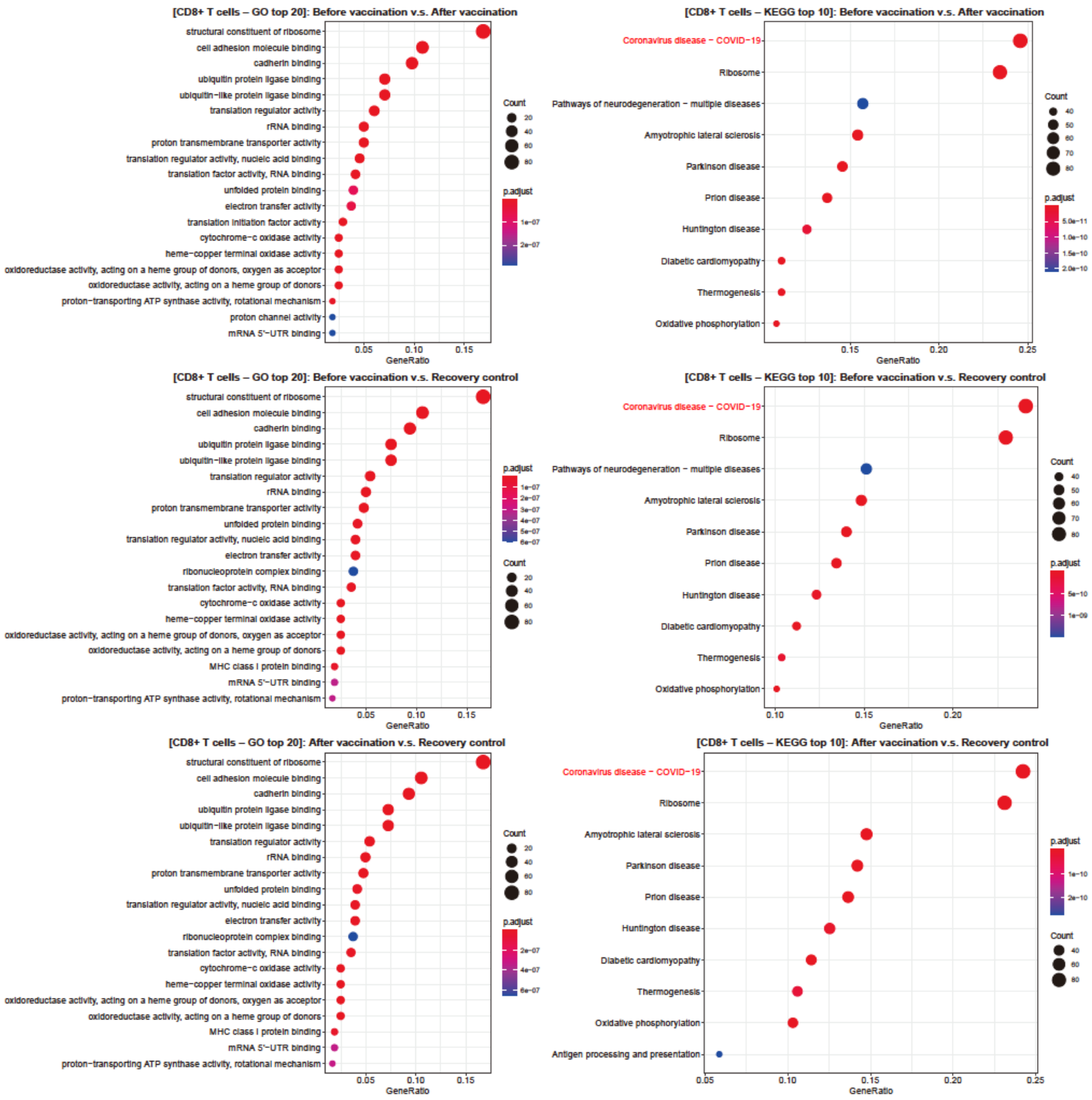

Figure S14. Enrichment GO (left panels) and KEGG (right panels) analyses for CD8+ T cells.

Figure S15

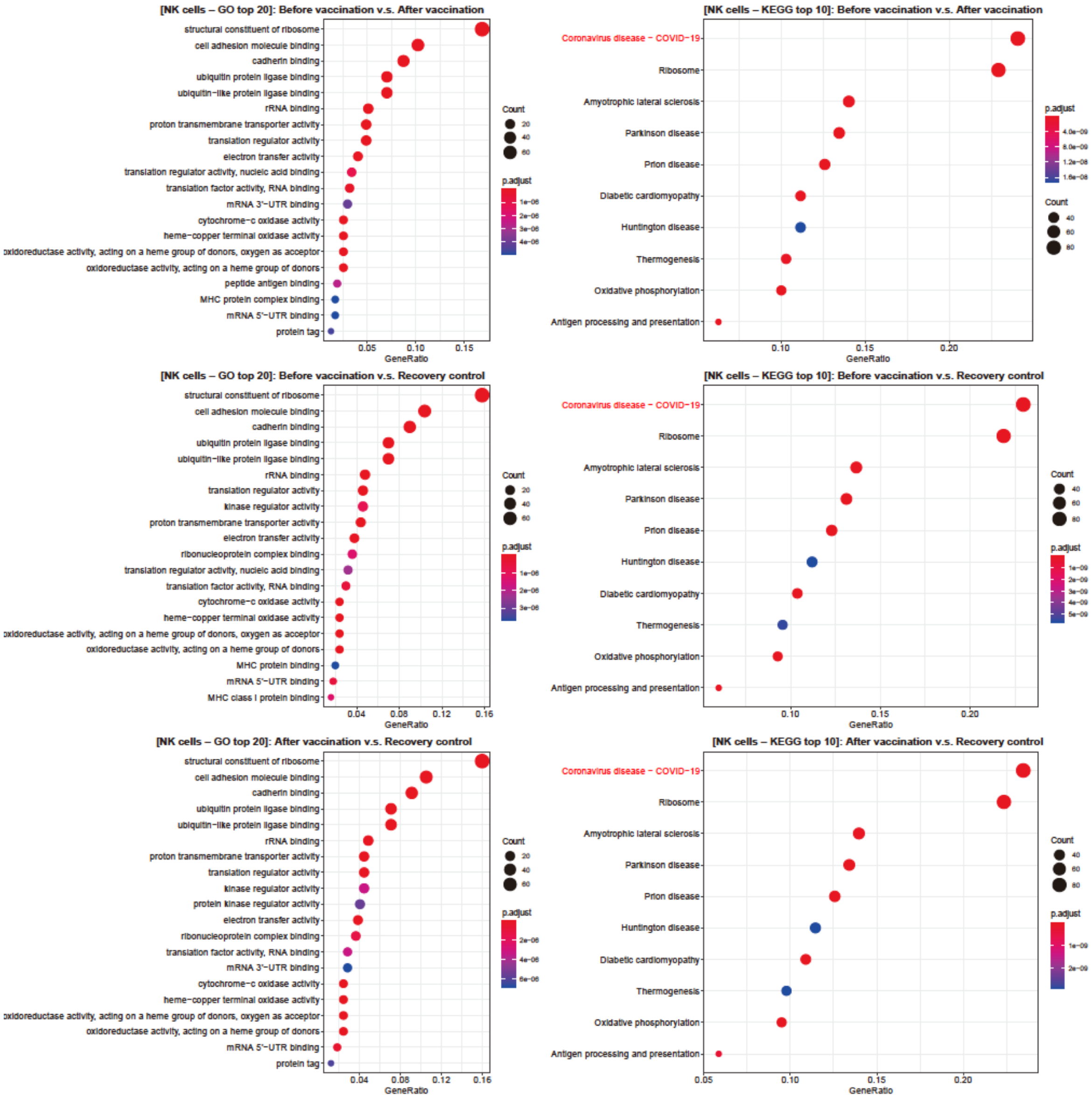

Figure S15. Enrichment GO (left panels) and KEGG (right panels) analyses for NK cells.

Figure S16

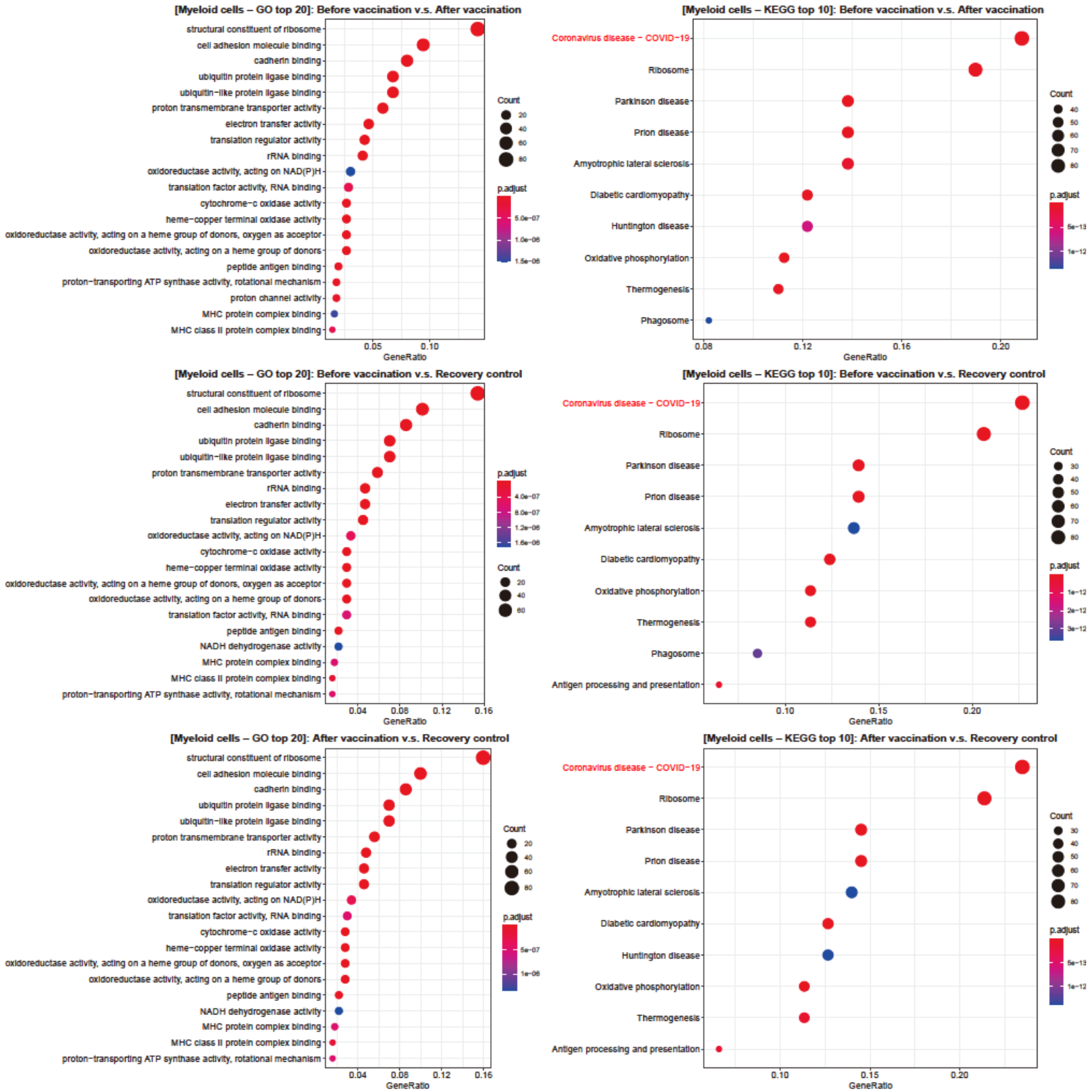

Figure S16. Enrichment GO (left panels) and KEGG (right panels) analyses for myeloid cells.
